# Oral Biofilm and Gender-Specific Health Considerations

**DOI:** 10.7759/cureus.91289

**Published:** 2025-08-30

**Authors:** Gregori M Kurtzman, Robert A Horowitz, Richard Johnston, Lillie Lanphier

**Affiliations:** 1 Dentistry, Private Practice, Silver Spring, USA; 2 Periodontology, NYU College of Dentistry, New York City, USA; 3 Internal Medicine, Private Practice, New York City, USA; 4 Neurology, Weill Cornell Medicine, New York City, USA

**Keywords:** erectile dysfunction, oral biofilm, pregnancy-related gingivitis, preterm pregnancy, prostate cancer

## Abstract

Oral biofilm plays a central role in the development of periodontal and systemic diseases, with growing evidence highlighting significant gender-specific differences. Hormonal fluctuations in women, during puberty, menstruation, pregnancy, menopause, and with oral contraceptive use, may alter the composition and behavior of oral biofilm, increasing susceptibility to gingival inflammation and periodontal disease. Conditions such as polycystic ovary syndrome (PCOS), osteoporosis, and pregnancy-associated gingivitis further demonstrate the influence of endocrine factors on oral health. In men, higher rates of severe periodontitis are observed, potentially linked to testosterone-related immune responses and behavioral factors with associations to lower sperm counts, increased incidence of prostate cancer, and erectile dysfunction. These distinctions underscore the importance of considering sex-specific biology in both the prevention and management of oral and systemic diseases influenced by biofilm. This study reviews the connections between gender-specific health and oral biofilm.

## Introduction and background

**Figure 1:**
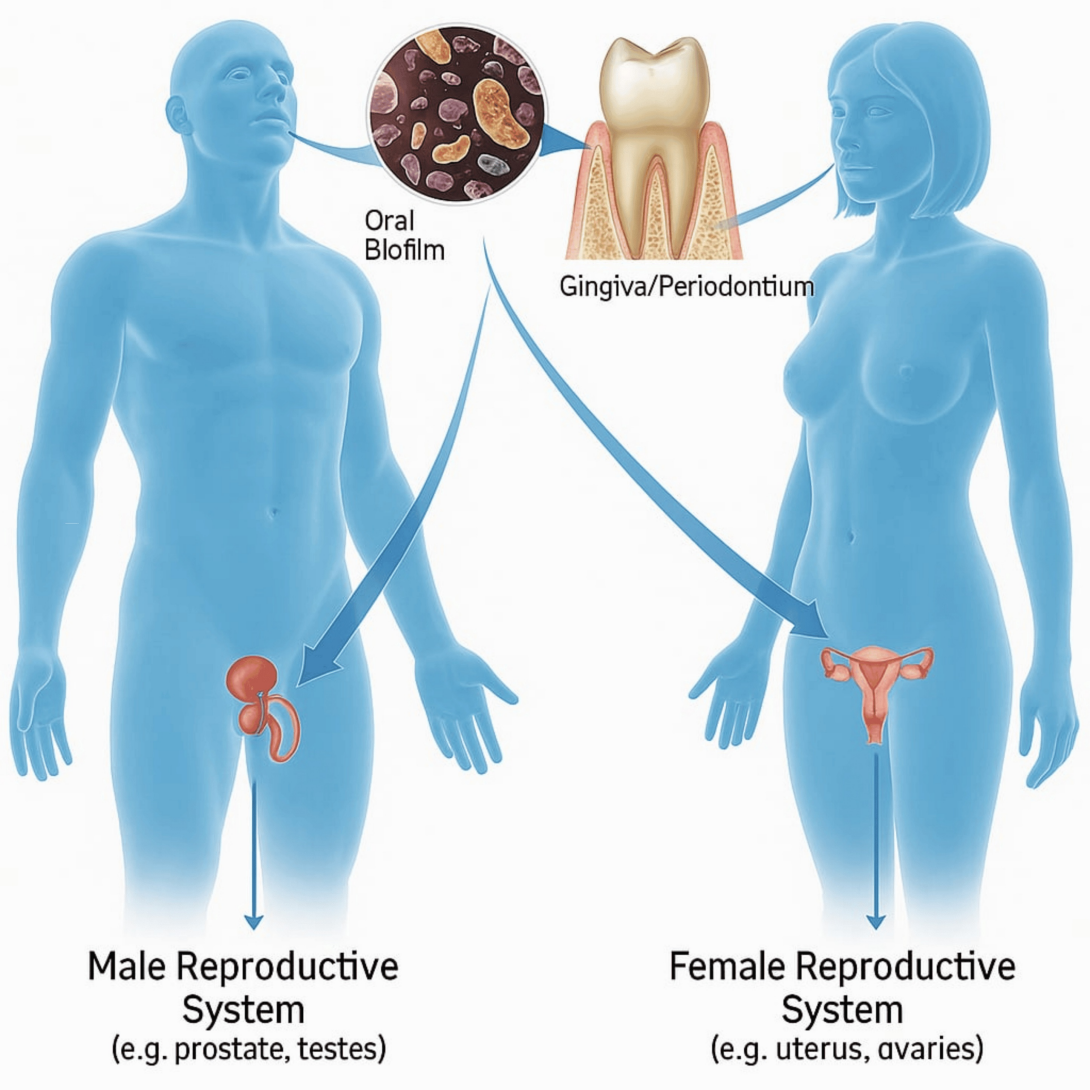
Bacteria in the oral biofilm have a connection to the reproductive systems in men and women, with transmission through the circulatory system to those distant systemic areas. This Illustration is created by the lead author, Gregori M. Kurtzman, of this study.

Oral biofilm, a complex microbial community, has been increasingly recognized not only as the primary factor in the development of periodontal and dental diseases but also as a contributor to systemic health conditions. The mechanisms of biofilm-related oral inflammation are well-documented, with increasing research suggesting the impact of oral biofilm may differ significantly between women and men, being influenced by biological sex, hormonal variations, and gender-specific behaviors (Figure 1).

In women, fluctuating hormone levels during puberty, menstruation, pregnancy, and menopause can alter the oral microbiome, increasing susceptibility to gingival inflammation and periodontal disease. Additionally, conditions such as polycystic ovary syndrome (PCOS), osteoporosis, and pregnancy-associated gingivitis highlight a unique interplay between systemic endocrine health and oral microbial ecology.

Men, on the other hand, tend to experience a higher prevalence of severe periodontal disease, potentially due to a combination of behavioral factors. Those behaviors include lower dental care utilization and biological differences in immune response. Moreover, associations between periodontitis and systemic conditions such as cardiovascular disease, metabolic syndrome, and erectile dysfunction raise important considerations for male-specific health risks tied to chronic oral inflammation.

This article explores the gender-specific implications of oral biofilm, with a focus on the differential immune, hormonal, and behavioral factors that influence oral-systemic health outcomes in women and men. Understanding these distinctions is critical for developing targeted preventive and therapeutic strategies that address the unique needs of each population.

Oral biofilm: a central player in oral and systemic disease

Oral biofilm, also referred to as dental plaque, is a densely structured, multi-species microbial community embedded within a protective extracellular matrix. The extracellular matrix is polymeric in nature and is of both host and bacterial origin. That matrix enables the bacteria to adhere to tooth surfaces and provides resistance to salivary flow, host immune mechanisms, and antimicrobial agents. This protective environment, referred to as “pathogenic synergism,” allows microorganisms to persist, adapt, and initiate disease processes both locally and systemically. The oral environment hosts over 700 bacterial species, with early colonizers such as *Streptococcus sanguinis* and *Streptococcus mutans *typically initiating adhesion to the tooth surface. Those are followed by more diverse organisms, including Actinomyces spp. and Veillonella spp., resulting in a complex and evolving microbial biofilm [1,2]. In a healthy state, this microbial ecosystem contributes positively to host defense by supporting mucosal immunity and maintaining a stable oral environment. This healthy state of stability and balance among various bacterial species contributing to health is called "microbial homeostasis," which, when disturbed, may lead to dysbiosis. However, shifts in microbial composition, referred to as dysbiosis, favor the overgrowth of pathogenic species, leading to chronic periodontal inflammation. This imbalance is recognized as a contributing factor to broader systemic issues, including cardiac, pulmonary, kidney, neurodegenerative conditions, and other systemic conditions. As the biofilm matures, it becomes more anaerobic, promoting colonization by periodontal pathogenic bacteria such as *Porphyromonas gingivalis*, *Tannerella forsythia*, *Treponema denticola*, and *Fusobacterium nucleatum *[3,4]. Those microorganisms are known to produce virulence factors that disrupt host tissues and evade host immune surveillance. Their encapsulation within the biofilm matrix renders them more resistant to antibiotics compared to free-floating (planktonic) forms [5,6]. Moreover, the interspecies interactions within the biofilm are not passive, as bacteria rely on metabolic cooperation and signaling pathways to survive and proliferate [7,8].

Quorum sensing, an interbacterial communication mechanism, regulates gene expression, biofilm development, and pathogenic behaviors, such as the release of enzymes and toxins [9,10]. Those signals also play a role in subverting host immune responses. Once host cells detect bacterial components, the immune system mounts a response involving neutrophils and macrophages. Chronic exposure to biofilm pathogens, however, often results in a prolonged and ineffective inflammatory reaction. Neutrophils that fail to eliminate bacteria within their short lifespan undergo cell death, releasing enzymes and reactive substances that can damage surrounding periodontal tissues [11,12]. The resulting inflammation contributes to gingival pocket formation, connective tissue loss, and alveolar bone destruction.

Beyond localized periodontal damage, oral biofilm contributes to systemic inflammation and has been implicated in conditions such as cardiovascular disease, diabetes, respiratory illness, osteoporosis, and numerous other health conditions [13,14]. Oral pathogens and their byproducts can enter the bloodstream via inflamed periodontal tissues, potentially influencing distant organs through immune activation and direct microbial invasion. Management of oral biofilm requires a combined approach involving both professional care, such as periodontal therapy and maintenance, and consistent daily oral hygiene practices by the patient. Individuals who maintain good oral health practices are significantly less likely to develop biofilm-related systemic complications compared to those with untreated periodontal disease [15,16]. Furthermore, the link between oral and systemic health is bidirectional. Patients with systemic health issues may have bacteria seeded from those systemic sites to the oral environment or increase oral inflammation, compromising the periodontal tissues, allowing increases in oral biofilm. This reciprocal relationship highlights the need for collaborative care models that integrate dental and medical management, particularly in patients with chronic systemic conditions.

Understanding the complex interplay between oral biofilm, host immunity, and systemic health is essential for developing more effective prevention and treatment strategies. As research continues to uncover these connections, personalized and interdisciplinary care will become increasingly important in improving patient outcomes.

## Review

Female puberty and gingival inflammation

The onset of puberty in females is accompanied by significant increases in circulating sex hormones, particularly estrogen and progesterone. These hormonal changes influence the gingival tissues and the host's immune response, heightening gingival sensitivity to local irritants. As a result, minimal amounts of oral biofilm can trigger an exaggerated inflammatory response, characterized by gingival enlargement, increased gingival bleeding, and crevicular fluid flow and microbial changes commonly referred to as puberty-associated gingivitis [17].

PCOS patients presented altered hormone levels, with elevated carbohydrate and lipid profiles. Additionally, those patients with PCOS present with more pronounced gingivitis than other patients. Those patients' neutrophils exhibit hyperactivity, which promotes interaction with the endothelium, potentially contributing to atherosclerotic disease [18].

Poor oral hygiene plays a central role in amplifying these conditions [19]. Inadequate plaque removal leads to an accumulation of oral biofilm along the gingival margin, acting as a persistent source of antigenic stimulation. In the pubertal female, the hormonal milieu alters the gingival vasculature, enhancing capillary dilation and permeability, which modulates immune cell activity. Thus, making the gingiva more reactive to those irritants. The synergy between hormonal sensitivity and microbial challenges results in gingival erythema, edema, and spontaneous bleeding. Moreover, elevated progesterone levels during puberty can promote the growth of specific periodontal pathogens, including *Prevotella intermedia*, which may utilize the patient's hormones as growth factors. When oral hygiene is poor, those bacteria thrive, further intensifying the inflammatory response.

Preventive care during puberty is critical. Emphasizing effective plaque control through regular brushing and flossing, along with professional dental cleanings and patient education, can significantly reduce gingival inflammation. Addressing oral hygiene habits early helps mitigate hormonally influenced gingival changes and reduces the risk of long-term periodontal complications. Additionally, early oral hygiene training aids in those home care skills becoming habits and influencing the patient's lifetime overall health.

Hormonal influence on gum health

Hormonal fluctuations throughout the female lifespan, particularly during puberty, menstruation, pregnancy, and menopause, have a significant impact on periodontal health [20]. Those endocrine changes can alter the vascular, immune, and microbial environments of the gingival tissues, often leading to periods of increased susceptibility to inflammation and periodontal disease. Estrogen and progesterone are the primary sex hormones implicated in modulating that gingival response. Estrogen affects the proliferation, differentiation, and keratinization of oral epithelial cells and plays a role in maintaining the structural integrity of the gingival epithelium [21]. Whereas progesterone increases vascular permeability and dilation, which enhances prostaglandin production, thus affecting the composition of subgingival microbiota. Collectively, this creates a gingival environment more prone to inflammation in response to even modest accumulations of oral biofilm [22]. 

During puberty, the rise in sex hormones leads to increased gingival sensitivity and an exaggerated inflammatory response. Similarly, during menstruation, some women may experience cyclical gingival changes, including periodic gingival swelling or bleeding. During pregnancy, elevated hormone levels, particularly during the second and third trimesters, may contribute to pregnancy gingivitis. In some cases, pyogenic granuloma formation may present, which often resolves postpartum but can be influenced by poor oral hygiene. Conversely, the decline in estrogen during menopause is associated with reduced salivary flow, altered oral microbial composition, and an increased risk for gingival atrophy and subsequent alveolar bone loss. Women who are postmenopausal may experience “menopausal gingivostomatitis,” which is characterized by easily bleeding, dry, and shiny gingiva [23].

Understanding the role of hormonal influence on oral health is essential for early intervention and personalized dental care. Preventive strategies should be tailored to each hormonal life stage. Emphasizing oral biofilm control, regular periodontal assessments, and coordination between medical and dental providers is essential when systemic hormonal therapies are involved. This integrated approach can help preserve oral health and improve quality of life across the patient’s lifespan.

Menstrual cycle and periodontal changes

The menstrual cycle, characterized by cyclical fluctuations in estrogen and progesterone, can exert a measurable influence on periodontal tissues [24]. Those hormonal variations affect gingival vasculature, connective tissue metabolism, and immune response, leading to transient changes in periodontal health that correspond to different phases of the cycle.

Progesterone levels peak and estrogen levels decline during premenstruation [25]. Many women experience increased gingival inflammation, referred to as menstrual cycle-associated gingivitis. This manifests clinically as gingival erythema, edema, and bleeding on probing, often in the absence of significant biofilm accumulation. Progesterone-induced increases in vascular permeability and prostaglandin synthesis result in a heightened inflammatory response, which amplifies tissue response to microbial irritants. Studies have also reported an altered neutrophil function and increased crevicular fluid flow during the luteal phase of the cycle, further contributing to local tissue inflammation [26]. Typically, those changes are reversible and can resolve following the onset of menstruation, but this underscores the importance of optimal oral hygiene maintenance during the menstrual cycle to minimize exaggerated periodontal responses [27].

Patients reporting cyclic bleeding or gingival discomfort during their menstrual cycle should be clinically evaluated by the dentist. Education on the interplay between hormonal cycles and periodontal health is essential, particularly for adolescent and reproductive-aged females, to encourage consistent oral hygiene practices and reduce the risk of chronic periodontal disease.

Menopause, oral biofilm, and bone loss

Menopause represents a significant physiological transition marked by a decline in estrogen production, having far-reaching effects on systemic and oral health. The reduction in estrogen levels during and after menopause contributes to changes in the periodontium, leading to increased susceptibility to oral biofilm accumulation, periodontal inflammation, and alveolar bone loss [28].

Estrogen has an important role in maintaining the integrity of the oral mucosa, vascular tone, and bone metabolism. Its deficiency results in decreased salivary flow, altered oral microbial composition, and thinning of the oral epithelium, which facilitates the proliferation of pathogenic bacteria within the oral biofilm. Those microbial changes, combined with a reduced mucosal barrier function due to thinning of the gingival tissue, increase the host's vulnerability to periodontal disease. A critical consequence of estrogen deficiency in postmenopausal women is the acceleration of alveolar bone resorption, similar to the mechanism observed in osteoporosis. That imbalance between osteoclastic and osteoblastic activity in the bone of the jaw leads to reduced bone density and structural deterioration (osteoporosis) [29]. When compounded by chronic periodontal inflammation related to persistent oral biofilm, an increase in the risk of attachment loss and tooth mobility is markedly noted. Females with osteoporosis presented with periodontitis in one study at 77.1% [30]. So, a preventive maintenance program for postmenopausal females, particularly osteoporotic females, who are at greater risk of tooth loss, could minimize those potential effects of bone loss on the teeth or implants if present. That decrease in oral bone density can be prevented and decreased utilizing low magnitude high frequency vibration (LMHFV) intraorally as part of the patient’s daily routine [31].

Management strategies to address menopausal hormonal oral tissue changes should include targeted periodontal care, rigorous biofilm control, and interprofessional collaboration with physicians managing hormonal or antiresorptive therapies. Adjunctive use of antimicrobial rinses, salivary substitutes, and hormone replacement therapy (when indicated) may provide additional benefits. Given the increasing aging population, understanding and addressing the oral-systemic implications of menopause is essential for preserving both periodontal and systemic bone health.

Oral contraceptives and gum health

The use of oral contraceptives, particularly those containing synthetic estrogen and progestin, can influence periodontal health by altering the host’s vascular and immune response to oral biofilm. Similar to hormonal fluctuations observed during pregnancy, puberty, and menopause, exogenous sex hormones introduced by oral contraceptives may increase gingival inflammation, even in the presence of minimal biofilm accumulation.

Estrogen and progestin in oral contraceptives promote increased vascular permeability and capillary dilation within the gingival tissues [32]. This hormonal influence can enhance the inflammatory response to local irritants such as those found in oral biofilm, leading to clinical manifestations such as gingival erythema, edema, and bleeding on brushing or probing. Some oral contraceptive formulations may modulate the composition of the oral biofilm microbiota, favoring the growth of pro-inflammatory bacterial species like *Prevotella intermedia* and yeast species (Candida), which utilize steroid hormones as growth factors [33]. The duration of oral contraceptive use appears to be an important factor. Long-term users show a higher prevalence of gingival inflammation and clinical attachment loss compared to non-users. The severity of those gingival changes has decreased in recent decades due to lower hormone concentrations in modern contraceptive formulations [34]. Thirty percent of women on oral contraceptives presented with localized or chronic periodontitis, while widespread aggressive disease was reported in 60% of participants in one study [35].

Importantly, oral hygiene care plays a role in those outcomes. Women using oral contraceptives with poor biofilm control are at a significantly greater risk of developing hormonally exacerbated gingivitis or early periodontitis. In contrast, effective oral hygiene home care and regular dental care can mitigate those effects, supporting periodontal stability.

Pregnancy-related gingivitis and periodontitis

Pregnancy induces profound hormonal, immunological, and vascular changes that can significantly impact periodontal health [36]. Elevated levels of estrogen and progesterone, particularly during the second and third trimesters, can amplify the inflammatory response to oral biofilm. This predisposes pregnant individuals to pregnancy-related gingivitis and, in more severe cases, progression to periodontitis. When left unmanaged, pregnancy gingivitis can progress to periodontitis in susceptible individuals, especially in the presence of pre-existing periodontal disease. Pregnancy-associated periodontitis involves irreversible loss of periodontal attachment to the teeth and alveolar bone. This has been associated in some studies with adverse pregnancy outcomes such as preterm birth and low birth weight, likely due to systemic dissemination of pro-inflammatory mediators and bacterial endotoxins.

Pregnancy gingivitis is the most common oral manifestation during gestation, which is characterized by gingival erythema, edema, bleeding on brushing or probing, and increased gingival crevicular fluid. Those changes are primarily due to hormone-induced vascular permeability and a shift in immune regulation, which heightens host response to the oral biofilm’s microorganisms. Alterations in oral microbiota, such as increased levels of *Prevotella intermedia*, which uses progesterone as a growth factor, contribute to the dysbiotic environment.

Pre-eclampsia is the second most frequent direct source of maternal mortality. Periodontitis appears as a significant risk factor for pre-eclampsia [37]. Periodontal pathogens could diffuse through the bloodstream, inducing an inflammatory response in the placenta. Additionally, inflammatory molecules produced in response to periodontal pathogens may migrate through the bloodstream, leading to a placental inflammatory response [38].

Clinical management of pregnancy gingivitis includes early identification and treatment of gingival inflammation, ideally beginning with preconception care or during the first trimester. Non-surgical periodontal therapy, such as scaling and root planing, is considered safe during pregnancy, preferably in the second trimester, and is effective in reducing inflammation and microbial load. Education on oral hygiene practices, nutritional counseling, and coordination with obstetric care providers are essential components of comprehensive prenatal care.

Recognizing pregnancy as a critical window for periodontal intervention offers a dual opportunity, improving maternal oral health and potentially mitigating systemic risks for both the mother and the developing fetus. Maintaining oral health during the peak of hormone release, such as pregnancy, helps alleviate the symptoms of periodontal disease as well as reduce the risk of adverse pregnancy outcomes, although studies report conflicting results [39].

Preterm pregnancy issues

Preterm birth is defined as delivery before 37 completed weeks of gestation. This remains a leading cause of neonatal morbidity and mortality globally. This is associated with a range of short- and long-term complications, including respiratory distress syndrome, neurological developmental impairments, and an increased risk of chronic health conditions later in life [40]. The etiology of preterm birth is multi-factorial, involving genetic, environmental, infectious, and inflammatory pathways. Unsupported, unfavorable beliefs about oral health and dental care utilization are common among pregnant women and new mothers [41]. Increasing evidence supports the role of maternal systemic inflammation as a contributing factor in spontaneous preterm labor. Infections of the genitourinary tract, intrauterine infections, and subclinical inflammatory states can trigger the release of pro-inflammatory cytokines and prostaglandins [42]. These in turn may induce cervical ripening, membrane rupture, and uterine contractions. Notably, periodontal disease has emerged as a potential extrauterine source of such inflammation.

Periodontal pathogens and their byproducts, such as *Porphyromonas gingivalis*, can enter the systemic circulation, eliciting an immune response that affects distant tissues, including the placenta and amniotic cavity. Studies have identified an association between maternal periodontitis and an increased risk of preterm birth and low birth weight, although causality remains under investigation. Those findings underscore the importance of maintaining optimal maternal health, including oral health, throughout the pregnancy.

Prevention and early management of risk factors for preterm birth should include comprehensive prenatal care, screening for systemic infections, and addressing modifiable lifestyle and health behaviors [43]. The integration of dental assessments and periodontal care into obstetric protocols is essential. These offer a preventative benefit, especially for women with existing periodontal disease or other inflammatory conditions. This interdisciplinary approach can contribute to improved maternal-fetal outcomes and support the broader goal of reducing the global burden of preterm birth.

Periodontal disease and sperm quality

Emerging evidence has drawn attention to the potential systemic effects of periodontal disease beyond the oral cavity, including its association with male reproductive health [44]. Periodontal disease is characterized by chronic inflammation and bacterial infection of the supporting structures of the teeth. This may negatively influence sperm quality through systemic inflammatory pathways, immune modulation, and microbial translocation [45].

The pathogenesis of periodontitis involves a dysbiotic oral microbiota triggering a sustained host immune response, leading to elevated systemic levels of pro-inflammatory cytokines such as tumor necrosis factor-alpha (TNF-α), interleukin-6 (IL-6), and C-reactive protein (CRP). These inflammatory mediators may disrupt hormonal regulation and spermatogenesis, potentially impairing sperm motility, morphology, and concentration. Additionally, periodontal pathogens and their endotoxins, such as *Porphyromonas gingivalis*, have been detected in the systemic circulation, which may reach the male reproductive tract, contributing to local oxidative stress and altered seminal parameters. Clinical studies have shown a correlation between poor periodontal status and decreased sperm quality [46]. This includes increased sperm DNA fragmentation, which results in reduced fertilization potential. Periodontal-systemic interactions affecting reproductive health are supported by animal models and observational human data.

Routine periodontal evaluation should be considered in the broader assessment of male infertility, particularly in cases of idiopathic subfertility. Improving periodontal health through non-surgical therapy, antimicrobial interventions, and patient education may serve as an adjunctive strategy in enhancing male reproductive outcomes [47]. Further interdisciplinary research is warranted to elucidate the mechanisms linking periodontal inflammation with sperm dysfunction and to evaluate the impact of periodontal treatment on fertility outcomes in men.

Oral biofilm and testosterone-related immune function

Oral biofilm's interaction with host immune function is complex and can be modulated by systemic hormonal factors, including testosterone, a cholesterol-derived molecule [48]. As the principal male sex hormone, testosterone not only governs reproductive and metabolic functions but also exerts significant immunoregulatory effects that influence host responses to microbial challenges, including those arising from oral biofilm.

Testosterone has been shown to exert anti-inflammatory and immunosuppressive properties by modulating the activity of various immune cells. It reduces the production of pro-inflammatory cytokines such as TNF-α, IL-1β, and IL-6, and down-regulates the activation of macrophages and dendritic cells. While this immunosuppressive action may confer protection against excessive tissue-damaging inflammation, it may also impair the immune system’s ability to mount an effective response against pathogenic oral bacteria. Consequently, men with higher circulating testosterone levels may exhibit a blunted inflammatory response to oral biofilm, potentially allowing for deeper microbial invasion and progression of periodontal disease [49]. Conversely, low testosterone levels, such as those observed in aging, have been associated with elevated systemic inflammation and increased susceptibility to chronic inflammatory conditions, including periodontitis. Studies suggest a bidirectional relationship, where periodontal inflammation can disrupt endocrine balance and contribute to reduced testosterone levels, while testosterone deficiency may worsen periodontal breakdown by exacerbating the host inflammatory response [50].

Understanding the interplay between testosterone and the immune response to oral biofilm is critical for developing personalized approaches to periodontal care. Future research exploring testosterone replacement therapy and its effect on periodontal parameters may offer novel insights into endocrine-immune-microbial interactions in oral health. Integrating hormonal assessment into periodontal risk profiling could enhance disease prevention and management strategies in hormonally influenced populations.

Oral biofilm and prostate cancer

Recent research suggests a potential link between chronic oral infections, particularly periodontitis, and the development or progression of prostate cancer. When dysbiosis occurs within the oral microbiome, this triggers a chronic inflammatory response that may extend beyond the oral cavity, influencing systemic health. Prostate cancer and periodontitis share several biological mechanisms, most notably the role of chronic inflammation [51]. *Porphyromonas gingivalis*, *Fusobacterium nucleatum*, and other keystone periodontal pathogens have been implicated in stimulating systemic pro-inflammatory cytokines such as IL-6, TNF-α, and C-reactive protein. Those mediators may contribute to the tumor's microenvironment by promoting angiogenesis, DNA damage, and immune evasion, the hallmarks of cancer development. Observational studies have reported associations between periodontal disease and elevated serum prostate-specific antigen (PSA) levels, even in the absence of overt prostate pathology. This suggests that oral inflammation may serve as a contributory factor in prostatic tissue irritation or carcinogenesis. One possible mechanism involves the translocation of oral bacteria or their byproducts via hematogenous routes, leading to localized inflammation in the prostate gland.

Additionally, some bacterial species within oral biofilms produce virulence factors capable of disrupting epithelial barriers, inducing oxidative stress, and modulating androgen metabolism, which may play roles in prostate carcinogenesis. The oral-prostate axis remains an emerging area of investigation, but preliminary findings support the hypothesis that maintaining periodontal health could be a modifiable risk factor in prostate cancer prevention and management [52]. Given those connections, dental and medical professionals should consider integrated care approaches. Periodontal screening and treatment may hold value not only for oral health but also as part of comprehensive cancer risk reduction strategies [53].

Oral biofilm and erectile dysfunction

Recent attention focusing on a potential link between oral biofilm-induced inflammation and erectile dysfunction (ED) has been reported in the literature [54]. ED, a prevalent condition affecting vascular, neurologic, and hormonal pathways, is increasingly recognized as a vascular disorder with an inflammatory component. Similarly, periodontitis is a chronic inflammatory disease initiated by dysbiotic oral biofilm, which has systemic ramifications extending well beyond the oral cavity. Multiple epidemiological studies have demonstrated a statistically significant correlation between periodontal disease and ED. Men with moderate to severe periodontal disease have been observed to have a higher prevalence of ED compared to periodontally healthy individuals. One of the shared pathological mechanisms between these two conditions is endothelial dysfunction, a process fueled by systemic inflammation, oxidative stress, and impaired nitric oxide (NO) bioavailability [55].

Oral pathogens such as *Porphyromonas gingivalis *and *Treponema denticola*, central components of the dysbiotic oral biofilm, produce endotoxins and virulence factors that trigger systemic inflammatory cascades. This includes the upregulation of proinflammatory cytokines such as TNF-α, IL-1β, and CRP, which can all impair endothelial nitric oxide synthase (eNOS) activity. Diminished NO production compromises vasodilation of the penile vasculature, a critical mechanism required for achieving and maintaining an erection. Additionally, chronic oral infections may contribute to the acceleration of atherosclerosis, including in the penile arteries, further exacerbating ED. The microvascular compromise observed in ED may therefore not only be a consequence of traditional cardiovascular risk factors but also a result of chronic oral inflammation.

Studies have also demonstrated that periodontal therapy may lead to measurable improvement in erectile function, as assessed by International Index of Erectile Function (IIEF) scores, suggesting that oral health may be a modifiable factor in ED management [56]. Findings support a shared inflammatory pathophysiology and underscore the importance of interdisciplinary collaboration between medical and dental professionals. Recognition of the oral-systemic link provides a compelling rationale for routine periodontal assessment in patients presenting with erectile dysfunction, particularly those with concurrent cardiovascular or metabolic risk factors [57].

Biofilm control strategies and adjunctive therapies

Managing oral biofilm effectively requires a dual approach - consistent professional dental care combined with consistent patient-driven oral hygiene home care. While daily brushing and flossing remain foundational, this has anatomical limitations. Toothbrush bristles generally access only 3-4 mm beneath the gingival (gum) margin, and dental floss typically reaches just 1-2 mm into the sulcus. Additionally, oral irrigators often fail to adequately penetrate into deeper periodontal pockets [58]. As a result, biofilm can rapidly re-establish itself, often exceeding pre-cleaning levels within 48 hours of disruption [59,60]. To complement mechanical cleaning, chemical adjuncts, particularly oral rinses, have been developed to enhance biofilm disruption. Chlorhexidine gluconate remains a commonly used antimicrobial rinse with efficacy against early-stage biofilms. However, it is less effective against mature, nutrient-depleted biofilms and may impair gingival fibroblast function with extended use [61]. Stabilized chlorine dioxide-based rinses (e.g., CloSYS, OraCare) represent a more tissue-compatible alternative. These agents have demonstrated strong biofilm-disrupting activity without cytotoxic effects, even when used at concentrations up to 40 parts per million, levels considered safe in drinking water [62,63]. Clinical studies support their efficacy in reducing plaque accumulation, gingival inflammation, and microbial burden. To enhance delivery, brushing immediately after rinsing with chlorine dioxide can help disperse the agent more effectively into interdental and subgingival areas. Additionally, combining oral irrigators with chlorine dioxide rinses may further increase subgingival access.

Dental prostheses, such as full or partial dentures, also accumulate biofilm and pose a significant risk for aspiration-related infections, particularly among elderly or medically compromised individuals. Regular cleaning using a denture brush in combination with chlorine dioxide rinses may lower the risk of respiratory infections associated with oral biofilm in this population.

Photobiomodulation (PBM) is another promising adjunctive therapy commonly referred to as low-level laser therapy. PBM employs red to near-infrared wavelengths to penetrate soft tissues, providing an anti-inflammatory and antimicrobial effect in areas that mechanical tools often fail to reach [64,65]. Studies have demonstrated PBM’s potential to reduce pathogenic oral flora, decrease caries susceptibility, and provide therapeutic benefits for conditions such as temporomandibular joint (TMJ) disorders, muscle soreness, oral lichen planus, and mucositis [66-68]. PBM may be especially beneficial for patients with physical or cognitive impairments who struggle to maintain adequate oral hygiene. Home-use devices such as the Accelite (Palm Beach Gardens, FL) have made PBM more accessible. This intraoral device delivers targeted light therapy through a bite-on applicator in short, daily sessions-typically five minutes, twice per day. For edentulous individuals, removing dentures during PBM use maximizes tissue exposure and therapeutic efficacy.

Together, these mechanical, chemical, and light-based strategies offer a comprehensive, evidence-supported approach to biofilm management. Integrating these modalities that are tailored to patient needs can enhance periodontal stability and reduce the systemic risks associated with chronic oral inflammation.

Discussion

Growing evidence underscores the significant role oral biofilm plays not only in the progression of periodontal disease but also in contributing to systemic health conditions, with distinct patterns emerging between genders. The chronic inflammatory burden triggered by biofilm-associated pathogens has been linked to a range of systemic disorders. Hormonal, behavioral, and immunological differences between men and women further shape those outcomes.

Sex-based differences in oral and systemic health reflect the complex interplay between hormonal regulation, immune response, behavior, and the oral microbiome. In women, fluctuations in sex hormone levels during key life stages are associated with notable shifts in the composition and activity of the oral microbiota. These endocrine changes can heighten inflammatory responses within gingival tissues, increasing susceptibility to gingivitis and periodontal disease. Various clinical conditions, such as pregnancy-associated gingivitis, polycystic ovary syndrome, and postmenopausal osteoporosis, underscore the bidirectional relationship between systemic endocrine function and periodontal status. Notably, estrogen deficiency has been implicated in altered bone metabolism and connective tissue remodeling, potentially contributing to alveolar bone resorption and progressive periodontal breakdown.

In contrast, men exhibit a consistently higher prevalence of moderate to severe periodontitis compared to women, a disparity attributed to both behavioral and biological factors. Delayed utilization of dental care, higher rates of tobacco use, and poorer oral hygiene practices are more commonly observed among male patients. Furthermore, gender-based differences in immune regulation may predispose men to more aggressive periodontal tissue destruction. Beyond oral implications, chronic periodontitis in men has been linked to an elevated risk of systemic inflammatory conditions, including cardiovascular disease, insulin resistance, prostate cancer, and erectile dysfunction. These associations suggest the presence of shared inflammatory pathways and reinforce the importance of periodontal health as a component of broader systemic disease prevention and management.

Gender-specific patterns highlight the importance of integrating oral health into systemic disease prevention strategies. Tailored approaches that consider hormonal influences, health behaviors, and gender-based biological differences are essential for effective prevention and management. Medical professionals should be attuned to these distinctions and collaborate with dental providers to deliver personalized care that addresses the broader systemic health implications of oral biofilm and periodontal disease.

A comprehensive biofilm management plan that incorporates routine dental professional care, individualized patient education, and improved home care therapies can mitigate systemic inflammation and improve overall health outcomes. Recognizing oral biofilm as a shared risk factor for both genders, but with distinct manifestations and consequences, reinforces the need for gender-informed strategies in both clinical practice and public health policy.

## Conclusions

Oral biofilm is increasingly recognized as a modifiable risk factor with systemic implications that differ between women and men. Gender-specific health conditions have been linked to chronic inflammation and microbial dysbiosis associated with periodontal disease. Those associations are mediated through systemic inflammatory responses, hormonal influences, and microbial translocation from the oral environment to distant systemic locations. Improved patient education and adherence to evidence-based oral homecare routines are key to disrupting oral biofilm and reducing the systemic inflammatory burden. However, patient efforts alone are insufficient. The dentist plays a critical role in the early identification and management of periodontal disease. Periodontal interventions should be considered integral to preventing biofilm-associated systemic complications.

Integrating oral healthcare into broader medical care is especially important when addressing gender-specific health risks. Collaboration between medical and dental professionals can aid in bridging gaps in care. This ensures that conditions influenced by oral inflammation are more effectively prevented or managed.
